# Intravascular extension of uterine sarcoma: A rare presentation mimicking venous thromboembolism

**DOI:** 10.1016/j.radcr.2026.04.033

**Published:** 2026-05-14

**Authors:** Amal Akammar, Younes Abdourabbih, Ismail Oughebbi, Hajjar Ouazzani Chahdi, Layla Tahiri Ousrouti, Ismail Chaouch, Nizar El Bouardi, Meriem Haloua, Badreeddine Alami, Moulay Youssef Alaoui Lamrani, Mustapha Maaroufi, Meriem Boubbou

**Affiliations:** a Mother and Child Radiology Department, Hassan II University Hospital Fez, Sidi Mohamed Ben Abdallah University, Fez, Morocco; b Department of Central Radiology, Hassan II University hospital Fez, Sidi Mohamed Ben Abdallah University, Fez, Morocco; c Department of Cardiovascular Surgery, Hassan II University Hospital Fez, Sidi Mohamed Ben Abdallah University, Fez, Morocco; d Anatomopathological Laboratory Department, Hassan II University Hospital Fez, Sidi Mohamed Ben Abdallah University, Fez, Morocco

**Keywords:** Uterine leiomyosarcoma, Intravenous leiomyomatosis, Intracardiac extension, Inferior vena cava, Computed tomography, Pulmonary embolism

## Abstract

Uterine leiomyosarcoma (ULMS) is a highly aggressive mesenchymal malignancy. While hematogenous spread is common, malignant intravenous leiomyosarcomatosis (IVLS) with extension into the heart is an exceptional and life-threatening phenomenon that presents significant diagnostic and therapeutic challenges. A 57-year-old postmenopausal woman presented with a voluminous pelvic mass. Initial MRI identified a high-grade ULMS with extensive local invasion and tumor thrombosis of the left ovarian vein. Following neoadjuvant chemoradiotherapy and total hysterectomy, the patient developed acute respiratory distress. Emergency echocardiography and CT angiography revealed a massive tumor thrombus extending from the iliac veins and inferior vena cava (IVC) into the right atrium, complicated by bilateral lobar pulmonary embolism. Despite an emergency beating-heart thrombectomy and intensive care support, the patient succumbed to refractory cardiogenic shock. This case underscores the critical role of multi-parametric MRI and CT angiography in identifying malignant intravascular extension. Differentiating tumor thrombi from bland venous thromboembolism is essential, as IVLS requires aggressive, multidisciplinary surgical management. Systematic vascular screening should be mandatory in cases of aggressive uterine malignancies to prevent fatal embolic events.

## Introduction

Uterine leiomyosarcoma (ULMS) is a rare and aggressive mesenchymal malignancy, accounting for less than 1% of all female genital tract cancers [1,2]. Characterized by high recurrence rates and a poor prognosis, ULMS typically disseminates via hematogenous routes, most frequently to the lungs, liver, and bones [3]. While local pelvic invasion is common, a highly unusual and life-threatening pattern of spread involves direct intravenous extension, a phenomenon termed intravenous leiomyosarcomatosis (IVLS). Unlike its benign counterpart, intravenous leiomyomatosis, malignant intravascular extension in ULMS is exceptionally rare, with only a few cases documented in the literature. This condition involves the migration of tumor thrombi through the uterine and iliac veins into the inferior vena cava (IVC), potentially reaching the right heart chambers.

Clinically, IVLS often mimics venous thromboembolism, leading to significant diagnostic challenges [3]. Multi-modality imaging, particularly MRI and CT angiography, is crucial to differentiate tumor thrombi from bland cruoric clots and to map the cephalad extent of the disease [[4], [5], [6]]. Accurate preoperative diagnosis is essential for planning complex multidisciplinary surgical interventions.

In this report, we describe a rare case of ULMS with intravenous extension reaching the right atrium, emphasizing the vital role of advanced imaging in managing this oncological emergency.

## Case presentation

A 57-year-old postmenopausal woman (G3P3), with no significant medical history, presented with a 3-month history of worsening pelvic pain. She reported no metrorrhagia or associated systemic symptoms. Clinical examination revealed a large, immobile pelvic mass. Vaginal examination confirmed an enlarged uterus, a firm cervix, and blunt vaginal cul-de-sacs, while rectal examination demonstrated bilateral parametrial induration. Initial pelvic ultrasound identified a voluminous, poorly-defined uterine mass. At the time of consultation, an initial pelvic ultrasound was performed, identifying a voluminous, poorly-defined uterine mass with significant internal vascularity on Color Doppler, suggesting a highly proliferative process.

**Fig. 1:**
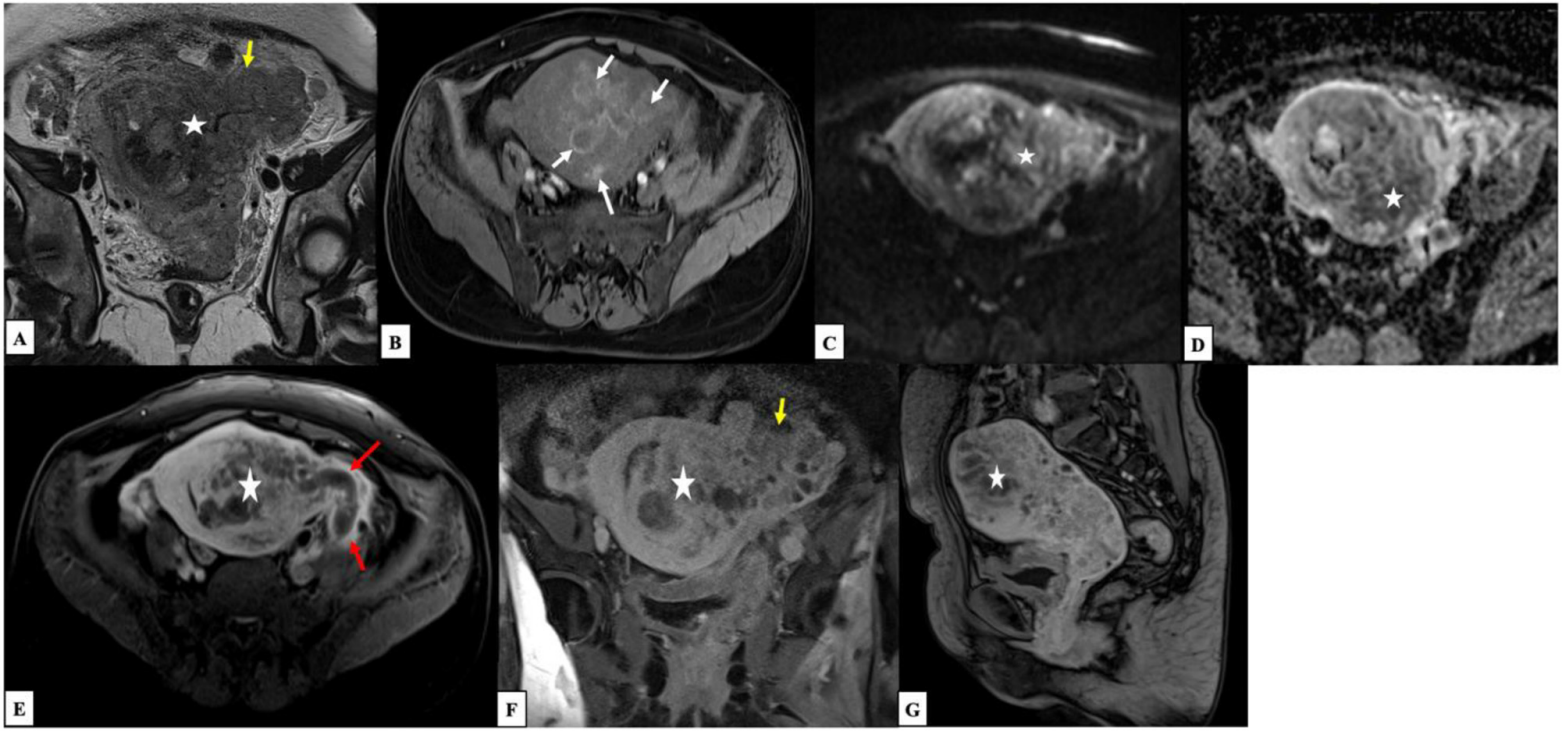
Axial MRI shows a substantial anterior myometrial tumor with irregular margins (star), exhibiting variable T2-weighted hyperintensity (A) with hemorrhagic areas showing high T1 signal intensity (white arrows), central necrotic areas, and significant restricted diffusion on diffusion-weighted imaging (C) with low ADC values (D). Post-contrast sequences demonstrated intense, heterogeneous enhancement (E, F, G). The tumor showed extensive local invasion into the endometrium, exocervix, the upper two-thirds of the vagina, and the parametria. Notably, an extraserosal extension was identified on the left (yellow arrow), invading the adnexa and resulting in tumor thrombosis of the left ovarian vein (red arrow).

A pelvic MRI was subsequently performed 1 week later to further characterize the mass. It revealed a substantial anterior myometrial tumor with irregular margins, exhibiting variable T2-weighted hyperintensity with central necrotic areas and significant signal restriction on diffusion-weighted imaging (DWI). Post-contrast sequences demonstrated intense, heterogeneous enhancement. The tumor showed extensive local invasion into the endometrium, exocervix, the upper two-thirds of the vagina, and the parametria. Notably, an extraserosal extension was identified on the left, invading the adnexa and resulting in tumor thrombosis of the left ovarian vein (Fig. 1).

Following the initial imaging, a transvaginal biopsy of the uterine mass was performed by the gynecological team 2 days after the MRI. Histopathological examination confirmed the diagnosis of high-grade uterine leiomyosarcoma (ULMS). Within one month of the initial consultation, a contrast-enhanced Thoraco-Abdomino-Pelvic (TAP) CT scan was performed and the case was discussed at a Multidisciplinary Team (MDT) meeting. The CT scan did not identify any distant secondary localizations or visceral metastases at that time.

The patient’s case was discussed at a Multidisciplinary Team (MDT) meeting. Due to the extensive local invasion and the involvement of the venous system, the tumor was classified as primarily unresectable. The consensus led to the initiation of neoadjuvant chemoradiotherapy to achieve local tumor control and potential downstaging. The patient subsequently completed 6 cycles of neoadjuvant chemoradiotherapy over a 6-month period. A follow-up MRI conducted eight weeks after the completion of treatment, demonstrated a significant regression of the primary uterine tumor. Given this favorable radiological response, the surgical team decided to proceed with a total hysterectomy and bilateral salpingo-oophorectomy. The surgical intervention was performed approximately ten months after the initial consultation. In the immediate postoperative period, the patient was transferred to the Intensive Care Unit (ICU), where she developed sudden acute respiratory distress. An urgent transthoracic echocardiography (TTE) was performed, which identified a voluminous, mobile mass in the right atrium measuring 50 mm in its largest diameter.

**Fig. 2:**
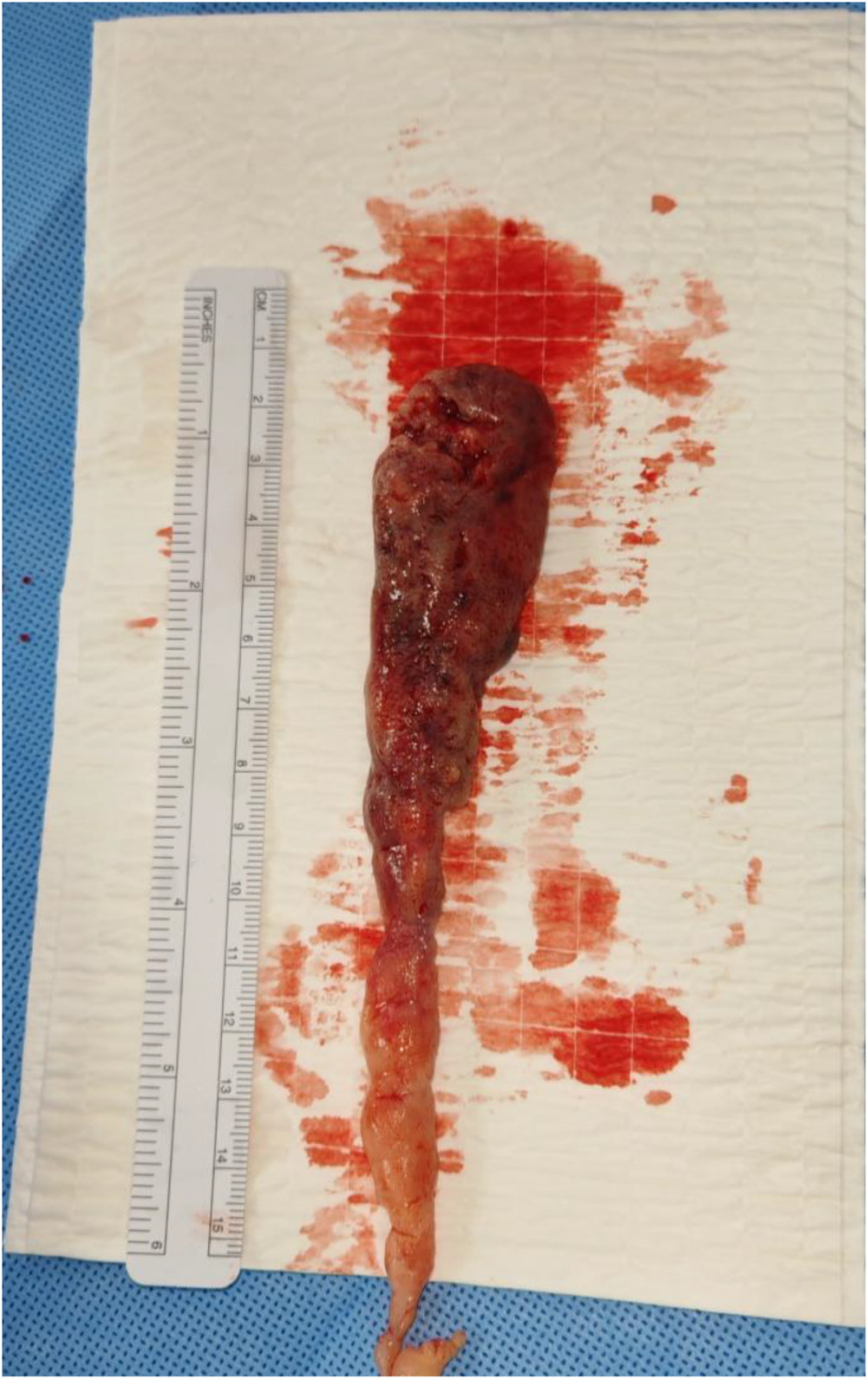
Gross appearance of the right atrial tumor thrombus following surgical resection.

Initially suspected to be a massive cruoric thrombus with an imminent risk of lethal pulmonary embolism, an emergency beating-heart thrombectomy was indicated. Intraoperatively, the surgical team identified a firm, solid tissue mass rather than the expected blood clot (Fig. 2). On the second postoperative day (POD 2), the patient underwent a contrast-enhanced thoraco-abdominal CT angiogram. The scan revealed proximal bilateral pulmonary embolism.

**Fig. 3:**
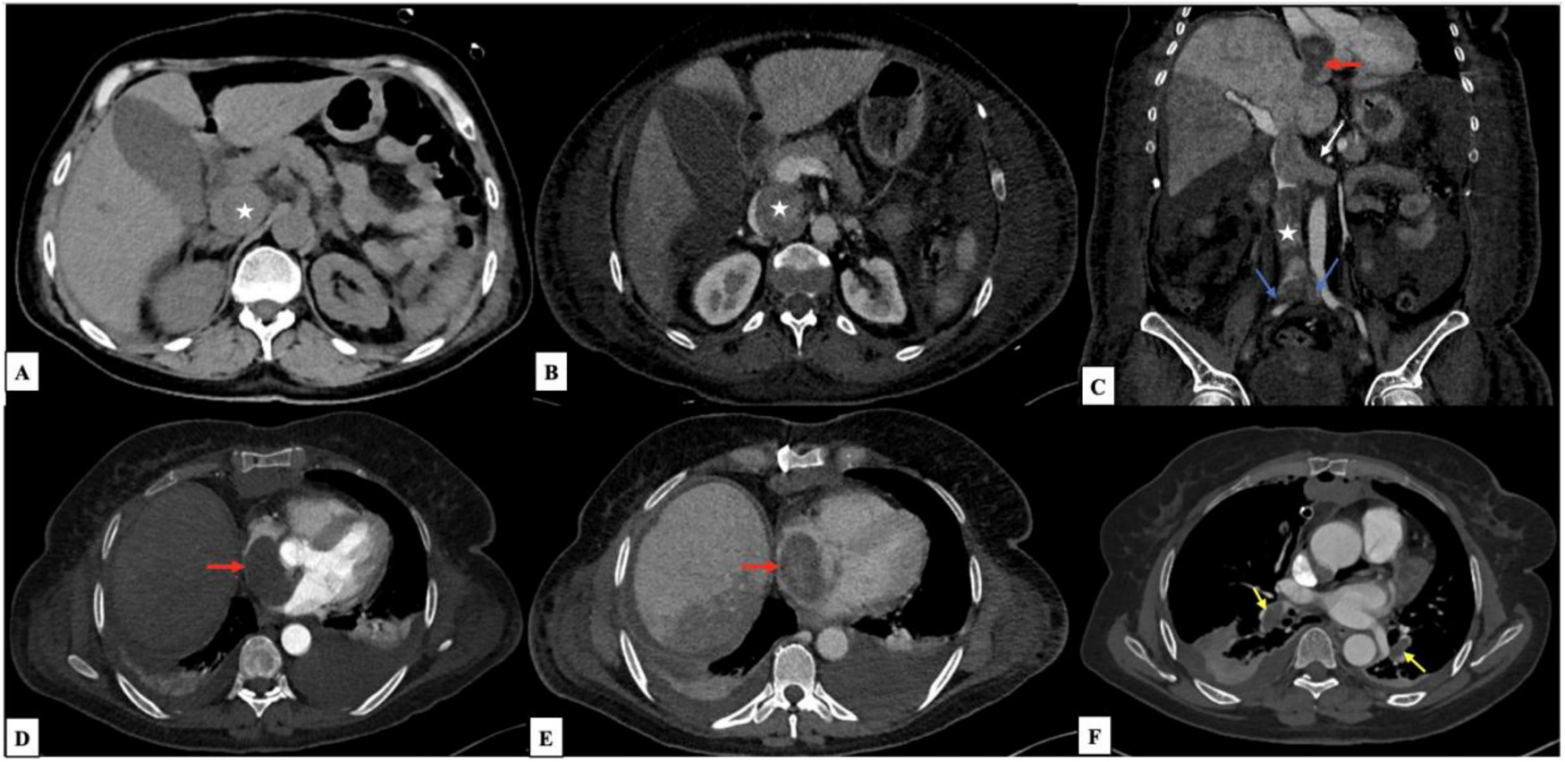
(A, B) Axial views showing a voluminous intraluminal soft-tissue lesion within the supra-renal inferior vena cava (star) with significant post-contrast enhancement. (C) Coronal reconstruction highlighting the extensive tumor mass with direct extension into the left renal vein (white arrow) and involvement of both common iliac veins (blue arrows), extending through the sub-renal and supra-renal IVC into the right atrium (red arrows). (D, E, F) Axial images at the level of the heart and lower lobes, identifying the intracardiac mass within the right atrium (red arrows) and bilateral inferior lobar pulmonary emboli (yellow arrows).

Imaging identified a voluminous intraluminal soft-tissue lesion within the inferior vena cava (IVC), involving both the supra- and sub-renal segments, with direct extension into the left renal vein and the right atrium. This intravascular mass demonstrated significant post-contrast enhancement. Additionally, bilateral inferior lobar pulmonary emboli were identified. The presence of both the intracardiac tumor extension and the bilateral lobar pulmonary emboli explained the patient's acute respiratory distress in the ICU. These findings confirmed the diagnosis of intravenous uterine leiomyosarcomatosis (IVLS) complicated by massive tumor extension and secondary pulmonary embolic events (Fig. 3).

**Fig. 4:**
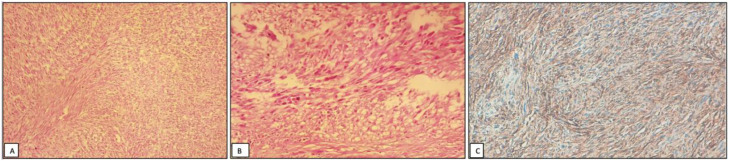
(A) Histopathological examination showing a spindle cell tumor with a fascicular growth pattern (HES x100). (B) Proliferation of atypical spindle cells with nuclear pleomorphism (HES x200). (C) Immunohistochemistry demonstrating strong and diffuse positivity for Desmin (x200).

Subsequent histopathological analysis of the resected specimen confirmed that this mass was a metastatic extension of the high-grade uterine leiomyosarcoma (ULMS), confirming the diagnosis of intravenous leiomyosarcomatosis (IVLS) reaching the heart (Fig. 4).

Despite the initiation of aggressive therapeutic measures, including therapeutic-dose anticoagulation with intravenous heparin and emergency surgical intervention, the patient’s clinical status continued to deteriorate. On the third postoperative day (POD 3), the patient developed refractory cardiogenic shock, secondary to the combined effects of the intracardiac tumor burden and the massive bilateral pulmonary embolism. Despite maximal intensive care support, the patient passed away. This rapid clinical decline underscores the malignant nature and the poor prognosis associated with intravenous uterine leiomyosarcomatosis when it reaches the heart and pulmonary vasculature.

## Discussion

Uterine leiomyosarcoma (ULMS) represents the predominant histological subgroup of uterine sarcomas, characterized by high aggressiveness and a poor prognosis. While the vast majority originate de novo, sarcomatous transformation of a benign leiomyoma occurs in only 0.2% of cases [1]. While distant metastases typically occur via hematogenous spread to the lungs or liver, the specific phenomenon of malignant intravenous extension “intravenous leiomyosarcomatosis (IVLS) “is an exceptional clinical entity [3,7]. Our case is particularly significant as it demonstrates the full spectrum of IVLS, spanning from the left ovarian vein to the inferior vena cava (IVC) and culminating in right atrial involvement.

The clinical challenge of IVLS lies in its ability to mimic benign conditions. As seen in our patient, symptoms are often non-specific (pelvic pain, abdominal mass), and intravascular lesions are frequently misdiagnosed as bland cruoric thrombi [3]. This leads to a critical diagnostic trap: treating a patient for common venous thromboembolism when, in fact, they harbor a progressive tumor thrombus. The literature suggests that IVLS must be suspected in any patient with a history of uterine malignancy presenting with new-onset lower limb edema or obstructive vascular symptoms [7,8].

From a radiological perspective, this case underscores the superiority of multi-parametric MRI and CT angiography. Unlike benign leiomyomas, which are typically homogeneously hypointense, ULMS typically presents on MRI as a substantial, infiltrative myometrial mass with intermediate-to-high T2 signal intensity. The presence of central "star-shaped" necrosis or irregular hemorrhage is a major hallmark of malignancy [4,6].

The differentiation is further refined using Diffusion-Weighted Imaging (DWI). ULMS exhibits significant signal restriction with low Apparent Diffusion Coefficient (ADC) values, typically below 0.9 × 10−3 mm^2^/s, reflecting high cellularity [5,6]. On dynamic contrast-enhanced (DCE) scans, these tumors show early, disordered heterogeneous enhancement.

In the literature, differentiating between a tumor thrombus and a bland (cruoric) thrombus is recognized as a major diagnostic challenge, yet it is crucial for determining the appropriate therapeutic strategy. According to established radiologic criteria, the most reliable feature for identifying a tumor thrombus is neovascularization, which manifests as significant post-contrast enhancement on CT or MRI. Bland thrombi, being composed of fibrin and platelets without a dedicated blood supply, typically remain non-enhancing.

Furthermore, several studies emphasize the `expanding sign', where a tumor thrombus causes a localized expansion of the venous lumen (e.g., the inferior vena cava), whereas a bland thrombus usually conforms to the vessel's diameter without distending it. On MRI, diffusion-weighted imaging (DWI) has been reported as a highly sensitive tool; tumor thrombi generally exhibit low apparent diffusion coefficient (ADC) values due to high cellularity, a feature rarely seen in bland thrombi. Finally, the clinical response serves as an indirect diagnostic marker: while bland thrombi are expected to regress under therapeutic anticoagulation, tumor thrombi are characteristically refractory to heparin, as observed in reported cases of intravascular leiomyosarcomatosis [7].

The extension into the right atrium represents a surgical and oncological emergency. CT is superior for mapping this cephalad extension, which is vital for surgical planning, especially when the tumor involves the heart chambers [8,9]. A unique teaching point from our case is the persistence of the atrial mass post-hysterectomy. Initially identified as a simple thrombus on echocardiography, it was confirmed as a secondary ULMS lesion after a beating-heart thrombectomy. This emphasizes that in the context of ULMS, any intravascular or intracardiac mass must be considered malignant until proven otherwise.

Due to the rarity of IVLS, no standardized treatment guidelines exist. However, surgical excision remains the cornerstone of management to alleviate obstructive symptoms, reduce the risk of fatal pulmonary tumor embolism, and potentially confer a survival advantage [9].

The management of IVLS requires a coordinated multidisciplinary approach. Surgery remains the cornerstone of treatment, aiming for complete resection of both the uterine and endovascular components, often necessitating cardiopulmonary bypass for intracardiac extensions. While anticoagulation is frequently initiated to prevent secondary thrombosis, it is ineffective against the solid tumor thrombus itself, as seen in our patient. The role of neoadjuvant therapies is to achieve local control, though their efficacy on the aggressive intravascular component remains unpredictable, requiring prompt surgical planning [9].

## Conclusion

Intravenous leiomyosarcomatosis is a rare but critical manifestation of uterine leiomyosarcoma. This case highlights the necessity of systematic vascular screening using multi-parametric MRI and CT angiography to differentiate tumor thrombi from bland thrombosis. Early radiological recognition of cephalad extension is vital to avoid diagnostic delays and to plan the aggressive multidisciplinary surgical intervention required for these high-risk patients.

## Patient consent

Informed written consent was obtained from the patient’s next of kin for publication of the Case Report and all imaging studies in Radiology Case Reports.
